# Preliminary Findings on the Morphometric Characteristics of the Olfactory Bulb in the Cat

**DOI:** 10.3390/ani14243590

**Published:** 2024-12-12

**Authors:** Rui Alvites, Abby Caine, Giunio Bruto Cherubini, Artur Severo P. Varejão, Ana Colette Maurício

**Affiliations:** 1 Centro de Estudos de Ciência Animal (CECA), Instituto de Ciências, Tecnologias e Agroambiente da Universidade do Porto (ICETA), Rua D. Manuel II, Apartado 55142, 4051-401 Porto, Portugal; ruialvites@hotmail.com; 2 Departamento de Clínicas Veterinárias, Instituto de Ciências Biomédicas de Abel Salazar (ICBAS), Universidade do Porto (UP), Rua de Jorge Viterbo Ferreira, nº 228, 4050-313 Porto, Portugal; 3 Associate Laboratory for Animal and Veterinary Science (AL4AnimalS), 1300-477 Lisboa, Portugal; avarejao@utad.pt; 4 Cooperativa de Ensino Superior Politécnico e Universitário (CESPU), Avenida Central de Gandra 1317, 4585-116 Gandra, Portugal; 5 Dick White Referrals, Six Mile Bottom, Cambridgeshire CB8 0UH, UK; abby.caine@dwr.co.uk; 6 Department of Veterinary Sciences, Veterinary Teaching Hospital “Mario Modenato”, University of Pisa, Via Livornese Lato Monte, San Piero a Grado, 56122 Pisa, Italy; giuniobruto@me.com; 7 Centro de Ciência Animal e Veterinária (CECAV), Universidade de Trás-os-Montes e Alto Douro (UTAD), Quinta de Prados, 5001-801 Vila Real, Portugal; 8 Departamento de Ciências Veterinárias, Universidade de Trás-os-Montes e Alto Douro (UTAD), Quinta de Prados, 5001-801 Vila Real, Portugal

**Keywords:** olfactory bulb, cat, head conformation, morphometry, olfactory system, MRI, companion animals

## Abstract

The aim of this preliminary study was to morphologically and dimensionally characterize the cat’s olfactory bulb in the sagittal plane and to establish potential relationships with the cranial conformation, based on the study of in vivo MRI images. Midsagittal and transverse T2-weighted images of the head of 40 cats subjected to MRI were selected. For each animal, the skull index was calculated to classify the cranial conformation. Then, for the olfactory bulb, the angle was calculated, the orientation was determined, and the sagittal section area was measured. It was established that animals classified as brachycephalic have more compact olfactory bulbs, with smaller cross-sectional areas, ventral orientation and smaller angles established with the line that goes from the hard palate and the intercondylar notch of the foramen magnum. Animals classified as dolichocephalic have more globose and wider olfactory bulbs, dorsal orientation, and larger angles. Mesocephalic animals present an intermediate position. Males and younger adult animals have olfactory bulbs with larger cross-sectional areas than females and older animals. This work allows for the preliminarily characterization of the olfactory bulb in cats in the sagittal plane, and the correlations identified with other head structures open doors for the use of the bulb as an early indicator for the establishment of alterations of varied etiology.

## Simple Summary

The aim of this preliminary study in cats was to study the shape of the olfactory bulb, the brain structure responsible for processing smell, and to relate its morphology to the conformation of the skull in the sagittal plane. MRI images were selected, and the olfactory bulb was analyzed and measured. This evaluation determined that brachycephalic cats, with shorter and wider heads and a flattened snout, have more compact olfactory bulbs, smaller cross-sectional areas, ventral orientation, and smaller angles established with the line that goes from the roof of the mouth to the base of the skull, where the vertebral column connects to the head. Cats classified as dolichocephalic, with an elongated and narrow skull and a longer snout, have more globose and wider olfactory bulbs, dorsal orientation, and larger angles. Mesocephalic animals present an intermediate position. Males and younger adult animals have olfactory bulbs with larger cross-sectional areas than females and older animals. This preliminary work allows for the characterization of the olfactory bulb in cats, and the correlations identified with other head structures open doors for the use of the bulb as an early indicator for the establishment of alterations of a varied nature.

## 1. Introduction

The olfactory bulb (OB) is one of the components of the olfactory system and works as a point of communication between the olfactory receptors present in the epithelium of the nasal cavity and different regions of the brain [1]. Anatomically, they are part of the paleocortex and appear as a double bulbar structure, rostral to each cerebral hemispheres in a ventrocranial position regarding the frontal lobes [2]. Each OB is located over one of the ethmoid fossae of the cribriform plate, whose foramina serve as a crossing point to the filaments of the olfactory nerves, being separated by the crista galli of the ethmoid bone. Caudally, the OBs continue as the olfactory peduncles, which have fibers (the intermediate olfactory tract) that form the rostral commissure connecting both hemispheres [3]. The space that separates the OB from the rest of the brain has been tentatively called the olfactory fissure [4], a term that, although not yet registered in the respective *nomina anatomica*, has been used for the anatomical description of this region in both humans [5] and animals [6] due to its good descriptive nature. The OB is part of the main olfactory system, in which it functions as an integration center for the information contained in volatile odorants decoded in the receptors of the olfactory epithelium, being responsible for its transmission to higher cortical centers after its correct filtration and modulation [7]. It is understandable that in species highly dependent on olfaction for behavioral expression and physiological normality, such as dogs and cats, small changes in OB and olfactory acuity can translate into serious changes that are, even so, difficult to be clinically quantified. In cats, in particular, the olfactory system has a close relationship with the regions of the brain related to stress responses, which can severely impact and exacerbate behavioral patterns in this species [8]. The clinical importance of OB tends to be neglected in veterinary medicine, although it is well established in humans. It is known, for example, that in humans the OB is one of the first locations affected by the establishment of neurodegenerative diseases such as Alzheimer’s and Parkinson’s, where atrophy of this region is observed parallel to changes in olfactory acuity [9], depositional changes, and reduced density of other brain locations like the temporal lobe gray matter [10,11]. In companion animals, mainly dogs, similar functional and structural changes associated with the establishment of cognitive dysfunction syndrome have also been indicated [12,13,14], allowing to foresee that, as in humans, OB could be a useful early indicator in the diagnosis of this syndrome in animals. However, in companion animals, the relationship between the establishment of these clinical entities and changes in the position, shape or volume of the OB have not yet been unequivocally described. A myriad of metabolic and infectious diseases or even the development of neoplasms and the occurrence of head injuries are also capable of inducing olfactory functional abnormalities, but it is believed that these are more related to changes in the nasal cavity and in the brain regions responsible for olfactory processing, failing to establish relationships with direct OB affection due to its distinctive anatomical position within the skull [1]. Knowing that the OB and its physical and positional characteristics can potentially be affected by various diseases, places this structure as a sentinel and early indicator of the development of these processes, both physically and functionally.

An in-depth knowledge of the anatomical and morphological characteristics of OB in companion animals is essential to allow its correct identification, description, and confirmation of its normality or the presence of structural changes. Due to its anatomical position, the OB can only be approached in vivo by imaging techniques, either by CT or high-resolution MRI, the latter technique being ideal for detailed determination of its position and description of its shape and volume, using T2-weighted sequences for better definition [15]. In dogs, some characterization studies have been carried out that attempt to establish relationships between the position, shape, volume, angle, and orientation of the OB regarding the rest of the brain and the head conformation of the animal, with the shape of the OB appearing to have a direct relationship with the depth of the lamina cribrosa in different breeds [16] and having the potential to be used as an indicator of the likelihood of developing complications associated with Brachycephalic Obstructive Airway Syndrome (BOAS) [17]. It is well established, for instance, that brachycephalic breeds of dogs have a more compact OB with a ventral orientation compared to mesocephalic or dolichocephalic ones due to the modifications of the head skeleton [17,18], which can translate into reduced olfactory acuity. In the case of cats, imaging studies directly focused on the anatomical description of the OB are limited, and it is known that this structure tends to appear rostral to the brain in a similar way to that observed in dolichocephalic dogs. In brachycephalic cat breeds, as well as in dogs, the OB is also less protruding and occupies a more ventral position and orientation [19]. Considering the established variations observed in the dog’s OB depending on the different cranial conformations, the authors hypothesize that the same should occur in the cat. Still, there is a need to establish well-defined relationships between the head conformation of different cat head conformations and the anatomical, morphological, and positional characteristics of the OB in this species, that can serve as a starting point for the use of OB modifications as an early indicator of changes associated with the development of deviations of a varied nature, namely in neurodegenerative entities or metabolic diseases.

The aim of this preliminary work is to characterize the OB in cats regarding its shape, position and orientation and to identify possible correlations between these characteristics and the different head conformations in this species, using a sagittal plane approach. More broadly, this study aims to explore the potential of using feline OB to indicate early changes secondary to diseases of different etiologies and that affect this structure.

## 2. Materials and Methods

The medical records and imaging database of the Dick White Referrals Veterinary Center’s Diagnostic Imaging service was explored to identify cats that underwent a head MRI scan between 2019 and 2024. Exams were analyzed and just those in which sagittal and transverse T2-weighted images of the head were available were considered. Only clinical cases with a complete clinical history in which the animal owner signed an informed consent form authorizing the use of image records for research purposes were included. Informed consent was obtained at the time of the animal’s admission to the hospital and no ethical approval was obtained due to the retrospective nature of this study, and prior acquisition of written owner consent for patient data to be included in scientific studies.

The absence of physical changes in the head that could affect the OB was established as inclusion criteria, and if changes were identified but these did not directly affect the OB either due to its anatomical distance or etiology, the cases were also included. Conversely, the exclusion criteria established were the unavailability of a complete medical history, the lack of informed consent signed by the animal’s owner, or the identification of head changes directly affecting the OB.

The animals’ clinical history was reviewed, and information collected included age and sex, imaging findings identified, and established diagnosis. During this phase, the anonymity of the animal and its owner was guaranteed, and the breed was not considered when obtaining clinical data and images. Following the established inclusion and exclusion criteria, 40 cats were included in this study. The group comprised a total of 23 males and 17 females. The age of the animals ranged from 1 to 18 years (7.6 ± 4.6 years).

The MRI images were obtained with an Lucente 0.3T–Hitachi MRI device (Hitachi Medical Systems, Zug, Switzerland) and the following parameters were considered: slice thickness of 3 mm, 15 slices, matrix size of 512 × 512, field of view of 180 mm × 180 mm (32,400 mm^2^). The obtained images were converted into DICOM format and analyzed using commercially available software (Weasis v4.5.1) [20]. Images were examined by a single image reviewer who was informed about the purposes of the study and the measurements to be performed. The reviewer received the files to be analyzed without any information about the associated data (age, sex, reason for performing the MRI, and registered breed of the animal) so as not to influence the evaluation and application of the established methodology. In this way, the reviewer could apply the measurement methods to determine which conformational category each animal belongs to, without knowing the specific breeds included in each group of cranial morphology. The sagittal images were identified as those which included the entire head, from the nasal planum (rostrally) to the intercondylar notch of the foramen magnum (caudally), and in which the largest possible image of the cerebellum and interthalamic adhesion were observed. The transverse images selected were those in which it was possible to obtain the maximum interzygomatic distance.

Animals were divided into one of three classical head conformation categories, adapting the methodology used by [21] for the Skull Index (SI) determination, considering the Skull Width (SW) and Skull Length (SL) integrated in the following formula:

$$SI=\frac{SW}{SL}\times 100$$

**Figure 1:**
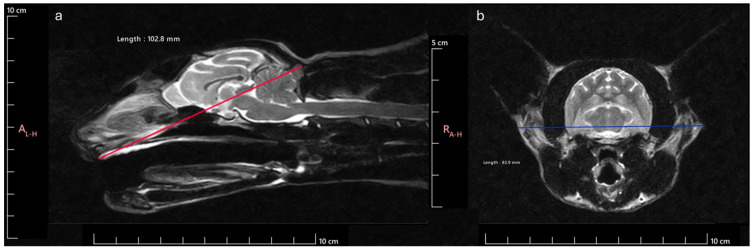
Cranial measurements necessary to determine the SI: (**a**) SL measurement: distance between the inion and the prosthion; (**b**) SW measurement: distance between the outer margins of the zygomatic arches.

SW was established as the maximum distance between the outer margins of the zygomatic arches and SL as the distance between inion (highest point of the external occipital protuberance, near the middle of the squamous part of occipital bone) and prosthion (rostral limit of the interincisive suture) (Figure 1). According to the SI obtained, the animals were classified as Brachycephalic (SI ≥ 80), Mesocephalic (SI 75–79), or Dolichocephalic (SI ≤ 75). Considering this division of animals regarding head conformation, 17 were classified as brachycephalic (8 males and 9 females), 9 as mesocephalic (6 males and 3 females) and, 14 as dolichocephalic (9 males and 5 females) (Table 1). The age of the animals was 8.53 ± 5.6 years for brachycephalic, 8.67 ± 4.15 years for mesocephalic and 5.86 ± 4.54 years for dolichocephalic. Thus, all animals in the different groups were skeletally mature, and no groups had significantly younger or older average ages.

**Figure 2:**
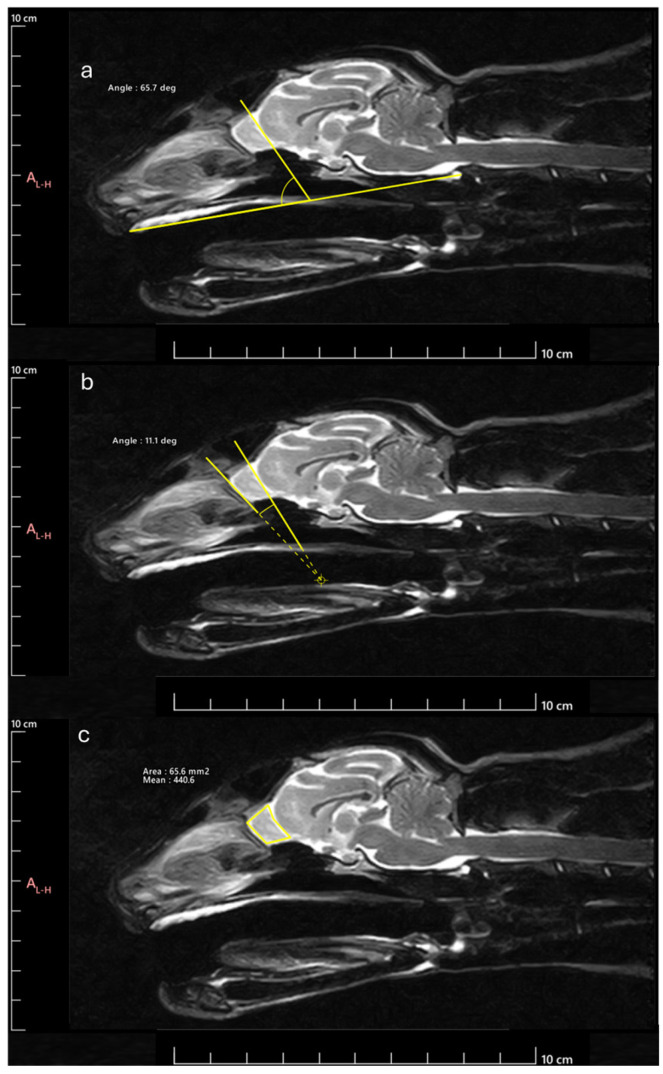
Methodology for determining the parameters considered: (**a**) OBA: angle formed by the line passing through the olfactory fissure and the line between the hard palate and the intercondylar notch of the foramen magnum; (**b**) OBO: open angle formed by the lines that pass through the olfactory fissure and the most rostral limit of the OB; (**c**) OBSA: automatically determined using the polygonal selection tool.

Next, the OB was measured to determine the following parameters: OB angle (OBA), OB orientation (OBO), and OB sagittal section area (OBSA). The OBA was calculated by applying the methodology described by [17]. Briefly, the angle formed by the line passing through the olfactory fissure and the line between the hard palate and the intercondylar notch of the foramen magnum was determined (Figure 2a). The OBO was evaluated considering the open angle formed by the lines that pass through the olfactory fissure and the most rostral limit of the OB (Figure 2b). Bigger angles were considered to correspond to OB with dorsal orientation and smaller angles to OB with ventral orientation. The OBSA was determined automatically using the polygonal selection tool of the selected software (Figure 2c), a tool that allows one to draw custom polygonal shapes over the MRI images to delineate regions of interest and to precisely measure variables such as the area after defining the vertices of the desired polygon, in this case, the limits of the OB in sagittal section. The same image reviewer carried out three measurements of all variables in all images on different days, with the average of the three measurements being considered in all cases. Whenever differences greater than 10% were observed between measurements of the same variable in the same animal, a new measurement was taken, and the 3 closest values were considered for the final average.

### Statistical Analysis

Statistical analysis was performed using GraphPad Prism software version 9.00 for Windows (GraphPad Software, La Jolla, CA, USA). The Normal Gaussian distributions of data were confirmed using the Shapiro–Wilk test. Data with normal distribution was compared using an Ordinary one-way ANOVA test, and data without normal distribution using a Kruskal–Wallis test. Linear regression analysis was performed to determine the Pearson correlation coefficients between variables. A value of *p* < 0.05 was considered statistically significant. The significance of the results is shown according to *p* values by the symbol *: * corresponds to 0.01 ≤ *p* < 0.05, ** to 0.001 ≤ *p* < 0.01, *** to 0.0001 ≤ *p* < 0.001, and **** to *p* < 0.0001.

## 3. Results

**Figure 3:**
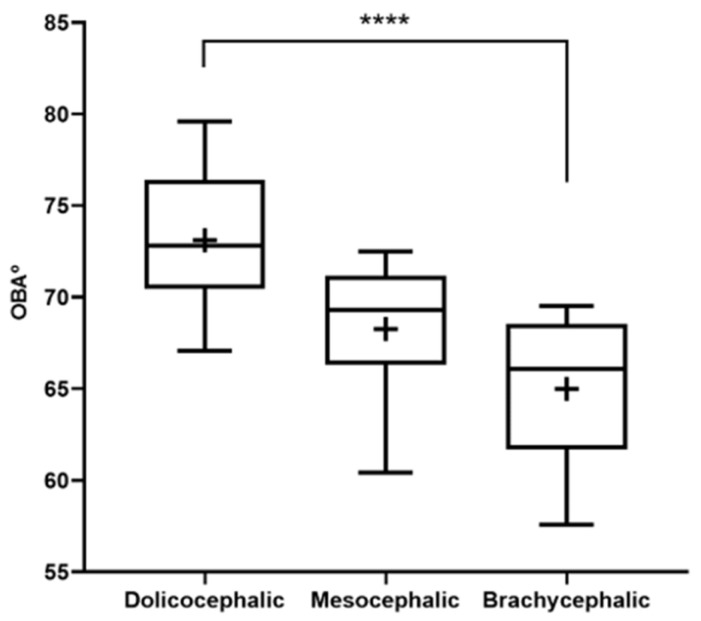
Box-and-whisker plots of the OBA determined from T2-weighted MRI reconstructions of the heads of 17 brachycephalic, 9 mesocephalic, and 14 dolichocephalic cats included in the study. The OBA was determined between the line passing through the olfactory fissure and the line running from the hard palate to the intercondylar notch of the foramen magnum. In each box, the middle line represents the mean value, and the lower and upper limits represent the 25th and 75th percentile, respectively. The whiskers represent the maximum and minimum values recorded. + represents the mean value. * corresponds to 0.01 ≤ *p* < 0.05, ** to 0.001 ≤ *p* < 0.01, *** to 0.0001 ≤ *p* < 0.001, and **** to *p* < 0.0001.

**Figure 4:**
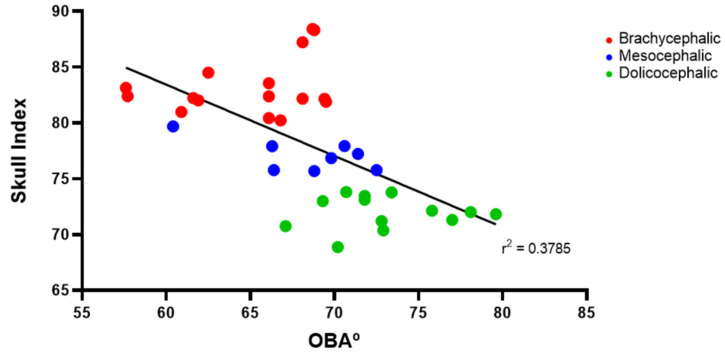
Scatter graphs of values of SI versus OBA determined from T2-weighted MRI reconstructions of 17 brachycephalic, 9 mesocephalic, and 14 dolichocephalic cats included in the study. The linear regression line is shown.

The results of the OBA determination can be found in Figure 3 and Table 2. The OBA is higher in the group of dolichocephalic cats and lower in the group of brachycephalic animals, with the group of mesocephalic animals occupying an intermediate position. The OBA values are 64.99 ± 1.01° for the brachycephalic group, 68.28 ± 1.37° for the mesocephalic group, and 73.12 ± 1.01° for the dolichocephalic group. Statistical differences are observed between the brachycephalic and dolichocephalic groups (*p* < 0.0001). Results of the linear regression analysis can be seen in Figure 4 and demonstrate that OBA significantly decreases with increasing SI, with a moderate negative correlation between the two variables (r^2^ = 0.38).

**Figure 5:**
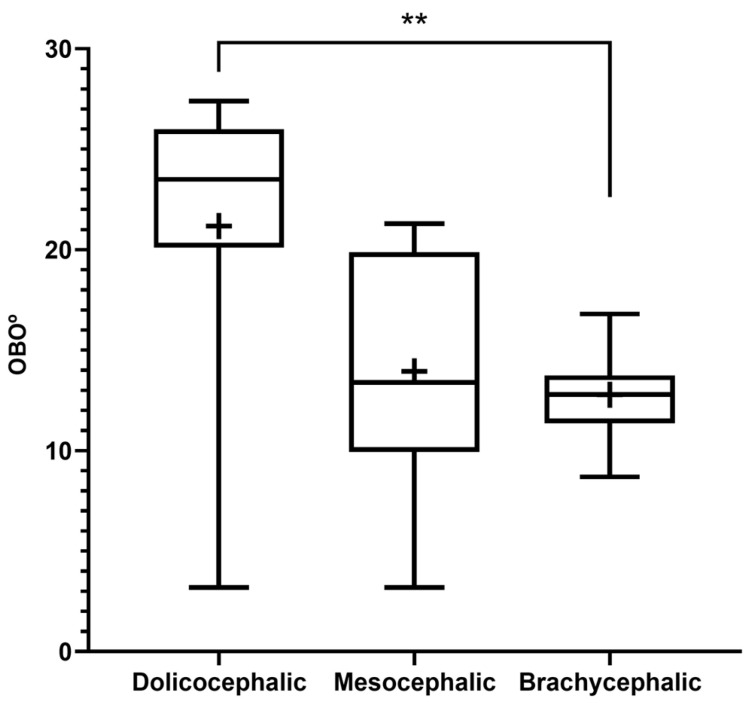
Box-and-whisker plots of the OBO determined from T2-weighted MRI reconstructions of the heads of 17 brachycephalic, 9 mesocephalic, and 14 dolichocephalic cats included in the study. The OBO was determined through the open angle formed by the lines that pass through the olfactory fissure and through the most rostral limit of the OB. In each box, the middle line represents the mean value, and the lower and upper limits represent the 25th and 75th percentile, respectively. The whiskers represent the maximum and minimum values recorded. + represents the mean value. * corresponds to 0.01 ≤ *p* < 0.05, ** to 0.001 ≤ *p* < 0.01, *** to 0.0001 ≤ *p* < 0.001, and **** to *p* < 0.0001.

**Figure 6:**
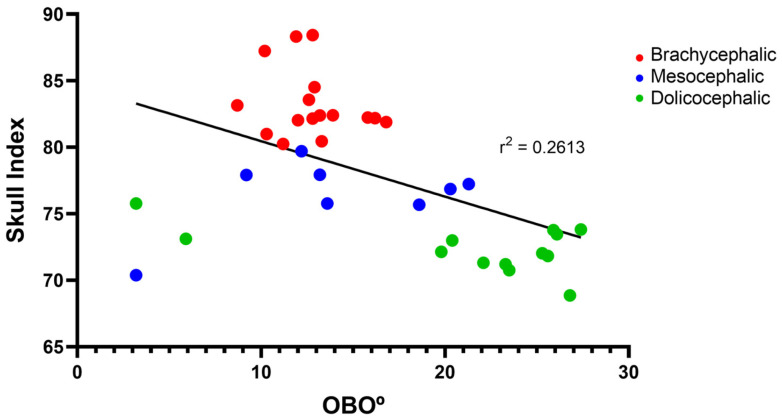
Scatter graphs of values of SI versus OBO determined from T2-weighted MRI reconstructions of 17 brachycephalic, 9 mesocephalic, and 14 dolichocephalic cats included in the study. The linear regression line is shown.

**Figure 7:**
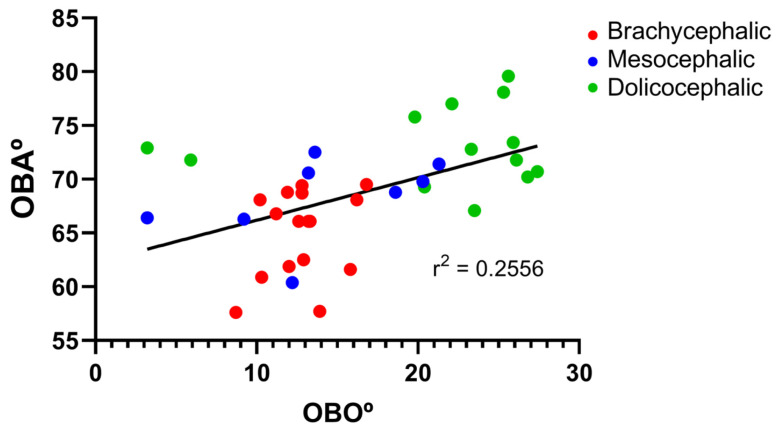
Scatter graphs of values of OBA versus OBO determined from T2-weighted MRI reconstructions of 17 brachycephalic, 9 mesocephalic, and 14 dolichocephalic cats included in the study. The linear regression line is shown.

The results of the OBO determination can be found in Figure 5 and Table 2. In the group of dolichocephalic animals, a larger open angle is observed (21.18 ± 2.15°), with these animals having a dorsal orientation of the OB. The group of brachycephalic animals presents an OB of ventral orientation associated with smaller open angles (12.79 ± 0.55°). The group of mesocephalic animals occupies an intermediate position (13.95 + 2.15°). Statistical differences are observed between the brachycephalic and dolichocephalic groups (*p* < 0.0024). Results of the linear regression analysis can be seen in Figure 6 and demonstrate that OBO significantly decreases with increasing SI, with a weak negative correlation between the two variables (r^2^ = 0.26). Additionally, the results of the linear regression analysis between OBA and OBO can be seen in Figure 7, which demonstrates, as expected considering the previous results, that a decrease in OBO is associated with an equal decrease in OBA, establishing a weak positive correlation between the two variables (r^2^ = 0.26).

**Figure 8:**
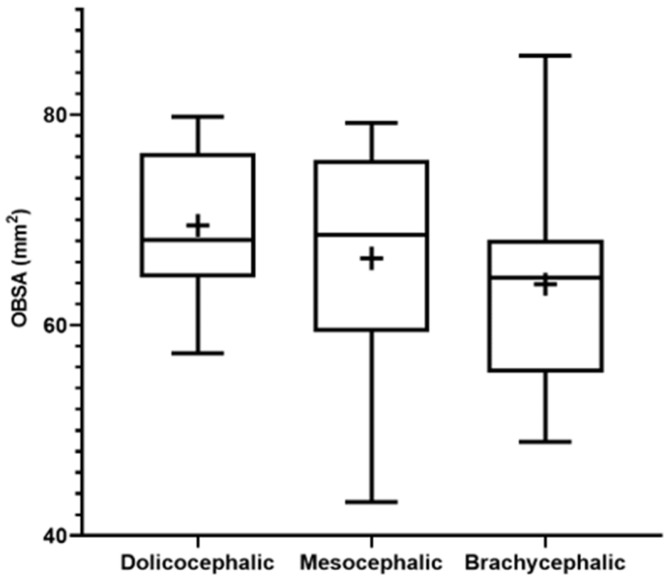
Box-and-whisker plots of the OBSA determined from T2-weighted MRI reconstructions of the heads of 17 brachycephalic, 9 mesocephalic, and 14 dolichocephalic cats included in the study. The OBSA was determined automatically using the polygonal selection tool of the used software. In each box, the middle line represents the mean value, and the lower and upper limits represent the 25th and 75th percentile, respectively. The whiskers represent the maximum and minimum values recorded. + represents the mean value. * corresponds to 0.01 ≤ *p* < 0.05, ** to 0.001 ≤ *p* < 0.01, *** to 0.0001 ≤ *p* < 0.001, and **** to *p* < 0.0001.

**Figure 9:**
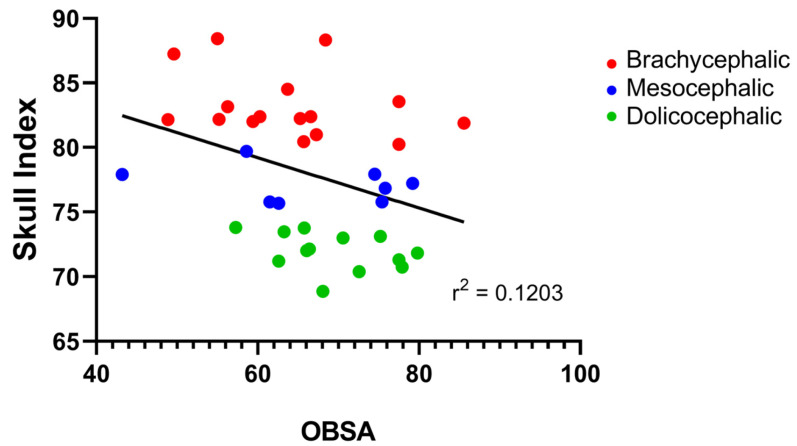
Scatter graphs of values of SI versus OBSA determined from T2-weighted MRI reconstructions of 17 brachycephalic, 9 mesocephalic, and 14 dolichocephalic cats included in the study. The linear regression line is shown.

The results of the OBSA can be found in Figure 8 and Table 2. In the group of dolichocephalic animals, the OB has a larger sagittal section area (69.48 ± 1.89 mm^2^), with the brachycephalic animals presenting the smallest area (63.89 ± 2.55 mm^2^) and the mesocephalic group occupying an intermediate position (66.35 ± 4.30 mm^2^). However, no statistical differences were observed between the groups. Results of the linear regression analysis can be seen in Figure 9 and demonstrate that OBSA significantly decreases with increasing SI, with a weak negative correlation between the two variables (r^2^ = 0.12).

**Figure 10:**
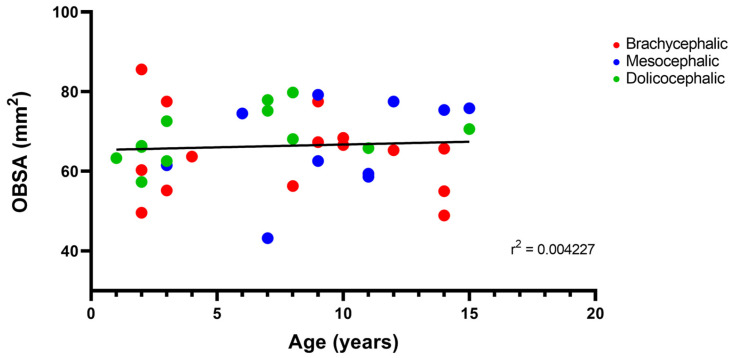
Scatter graphs of values of OBSA versus Age determined from T2-weighted MRI reconstructions of 17 brachycephalic, 9 mesocephalic, and 14 dolichocephalic cats included in the study. The linear regression line is shown.

The results of the linear correlation between OBSA and age can be seen in Figure 10, demonstrating an increase in the OB sagittal section area with advancing age, but with a very weak positive correlation between the two variables (r^2^ = 0.004).

**Figure 11:**
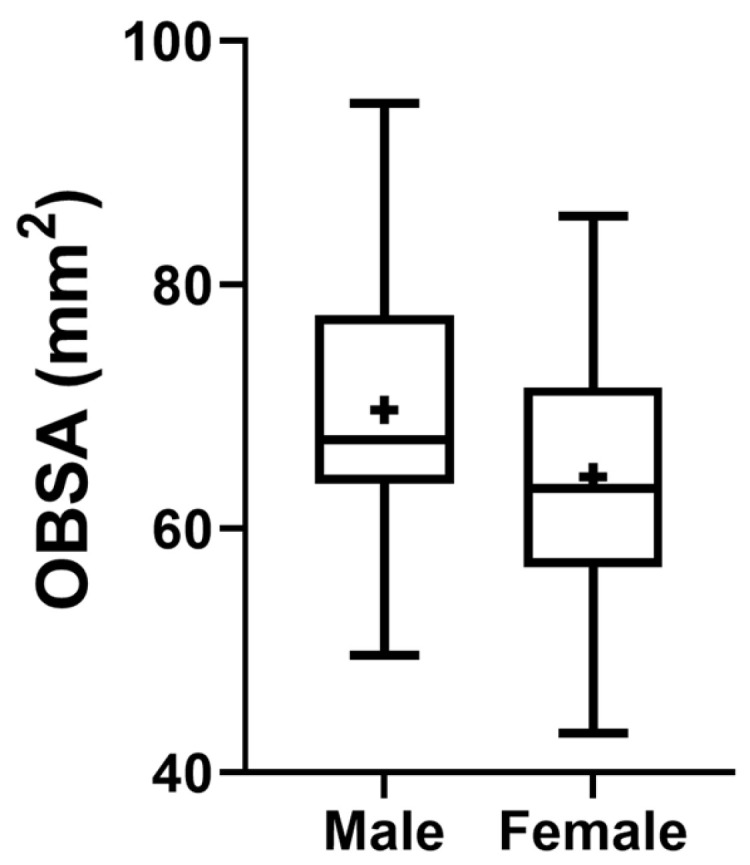
Box-and-whisker plots of the OBSA determined from T2-weighted MRI reconstructions of the heads of 17 brachycephalic, 9 mesocephalic, and 14 dolichocephalic cats included in the study. The OBSA was determined automatically using the polygonal selection tool of the used software. In each box, the middle line represents the mean value, and the lower and upper limits represent the 25th and 75th percentile, respectively. The whiskers represent the maximum and minimum values recorded. + represents the mean value. * corresponds to 0.01 ≤ *p* < 0.05, ** to 0.001 ≤ *p* < 0.01, *** to 0.0001 ≤ *p* < 0.001, and **** to *p* < 0.0001.

The OBSA results that consider the sex of the animals can be seen in Figure 11. The group of females presents a smaller sectional area of the OB compared to the male OB, although no statistical differences are observed between the two groups.

Combining all the results revealed that the OB of brachycephalic animals present a smaller OBA, with a ventral orientation, and a smaller sagittal cross-sectional area, in opposition to dolichocephalic animals that present larger OBA, tending to be dorsally oriented and a larger sagittal cross-sectional area. Mesocephalic animals occupy an intermediate position. Males present larger sagittal cross-sectional areas than females, with an increase in the sagittal cross-sectional area in older animals compared to younger ones. These results effectively demonstrate that, in addition to age and sex, cranial conformation and the consequent cranio-osseous modifications can affect the anatomical arrangement of intracranial central nervous system structures, in this case being able to influence the olfactory acuity of animals and allow the establishment of morphological and imaging characteristics of the OB related to the cranial conformation in which each breed of cat is inserted.

## 4. Discussion

In recent years, to a limited extent, efforts have been made to establish relationships between the morphological and dimensional characteristics of the OB, olfactory acuity, and the development of structural or functional changes in the brain or other regions of the head. In humans, this parallelism is becoming well established, and it is possible to relate the phenomena of bulbar atrophy to the development of neurodegenerative diseases and senility, the occurrence of cranial trauma, and the establishment of infectious and metabolic diseases, with the loss of olfactory acuity being a known consequence that is clinically easy to identify [22,23,24,25]. Similarly, evidence of a positive correlation between OB volumes and olfactory function has also been described [26].

In companion animals, superior olfactory ability is well established, both in dogs [27] and cats [28]. However, not only are studies carried out on cats more limited, but the influence of inter-individual variations depending on the breed, sex, or age of animals of both species ends up being rarely explored. In dogs, imaging studies to characterize the OB allow the creation of the first relationships between the characteristics of this structure and other elements of the brain and/or head, such as the cranial conformation or the soft palate [17,18]. This more in-depth knowledge of the OB [29] has also allowed to identify relationships between the dimensions and integrity of the OB and the establishment of diseases of different etiologies, such as neurodegenerative [30] or neoplastic [31]. Some studies have been carried out in cats to characterize the OB [32] and its recesses [33], but these remain limited.

This study represents a significant advancement in understanding the morphometric characteristics of the OB in cats, specifically through a sagittal plane approach. The sagittal plane approach offers distinct advantages for the morphometric analysis of the OB, providing direct and detailed visualization of its spatial relationships with critical surrounding anatomical landmarks, allowing for accurate assessment of OB orientation, angular measurements, and cross-sectional areas in relation to cranial and palatal structures. The methodology was rigorously designed, utilizing high-resolution T2-weighted MRI images and advanced measurement tools to ensure accuracy and minimize bias. Notably, the use of the polygonal selection tool enabled precise delineation and area calculations of the OB in sagittal sections, providing a robust framework for evaluating its dimensional characteristics. The sagittal approach is particularly suited for evaluating longitudinal features and detecting subtle morphological and alignment variations along the cranial axis that may not be apparent in transverse or coronal plane imaging, particularly when addressing small structures such as the OB in cats. Furthermore, its use enhances measurement reproducibility by simplifying anatomical alignment and minimizing variability as it avoids the potential inconsistency introduced by rotational or asymmetrical positioning inherent in transverse imaging. This methodology supports precise and consistent data acquisition, making it a robust framework for analyzing structural adaptations linked to cranial conformations and facilitating future studies on functional or pathological changes involving the OB, particularly in cats.

Head conformation refers to the shape and structure of the skull, a definition that can be applied to all species with the necessary adaptations. In companion animals, as a result of the selection processes and planned breed crossings [28,29], the evolution of the different categories of cranial conformation took place in a marked way, translating not only into evident differences in anatomy and external morphology but also influencing the behavior and physiological normality of different systems such as the nervous, digestive, respiratory and ocular in both dogs [30,31,32] and cats [33]. In the cat, although the division into the same categories is also made, the distinction between these is more difficult and there are no cut-off values as well defined as in the dog [34]. The dolichocephalic category includes the Siamese and Balinese; the mesocephalic ones include the Maine Coon or British Shorthair; Brachycephalic cats include Persians and Scottish Folds. Within brachycephalic breeds, there are still attempts at subdivisions, such as Dool-Face and Peke-Face in the case of Persian cats [14]. Just like dogs, in cats, the brachycephalic breeds are more prone to various medical problems [16,33,35]. Thus, in this study the methodology used by [16] was adapted to establish the SI limits that allowed the classification of cats as brachycephalic (SI > 80), mesocephalic (SI 75–79), or dolichocephalic (SI < 75). The standardized classification of skull types using SI further reinforced the reliability and relevance of the findings.

The results of the OBA determination follow the same trend as that observed in dogs, with brachycephalic animals presenting a lower OBA compared to dolichocephalic and mesocephalic breeds and with a negative correlation between the two variables. However, in dogs the established correlations are stronger than in cats. Even using a different methodology, it is also observed that, as in dogs, cat brachycephalic breeds tend to have a smaller open angle and OB with a ventral orientation compared to the dorsal orientation of dolichocephalic breeds, with a negative correlation established between the two variables. Once again, the correlation is stronger in dogs than in cats. It is important to note that cats have a larger cranial capacity, i.e., the volume of the interior of the cranium occupied by the brain, than dogs [34,35], and although classical divisions dependent on cranial conformation can also be established in cats, these variations are not as wide as those observed in dogs [36]. This may explain why, although in cats the influence of cranial conformation on the position, shape and arrangement of the OB is also observed; this marker influence has not been recorded in dogs. In any case, the use of a larger number of animals in this study could have allowed for more in-depth conclusions.

The OBSA was determined automatically using the polygonal selection tool of the selected software, allowing for the determination of the sectional area of the OB in the midsagittal images, although no statistical differences were observed that would allow deeper conclusions. The limited number of morphometric and dimensional studies in cats [32] do not allow, currently, to compare results, but it is expected that, as in dogs [18], the bone changes observed in brachycephalic skulls lead to a shortening of the nasal cavity with compression of the cribriform plate, which becomes flatter, leading to the OBs arranged on it also becoming more compact and less globose, contrary to what is observed in dolichocephalic animals. This compaction leads, in turn, to a smaller sagittal cross-sectional area. Similarly, the comparison of the transectional area of the OB between males and females also demonstrated a smaller OB sectional area in females, without statistical differences, a result that corroborates those observed in humans [37]. However, it is important to note that this result is not related to a better olfactory acuity in males, since a greater number of active cells in the OB was previously identified in females [37,38], this being a better functional indicator than the dimensional issue and attributing to females a greater capacity to retain long-time memory of odors. Likewise, there is evidence that the reproductive status and menstrual cycle of females may also influence olfactory acuity [39] and, potentially, the OB characteristics, and the reproductive status of the females included in this study were not considered.

Regarding the relationship between OBSA and age, a positive, albeit very weak, correlation was observed between the two variables, with older animals presenting a bigger sectional area than younger ones. This result may be in line with other evidence in animals and humans indicating that cellular activity in the OB and the capacity for long-time memory of odors is greater in adults than in juvenile animals [38], although OB volume tends to decrease in older adults [40], particularly in cases of neurodegenerative evolution [24]. There appears to be a relationship between cognitive maturation and olfactory capacity in both humans and animals [13,41], which explains the larger dimensions of the OB in adult cats compared to juvenile ones. However, it is important to note that maturation in animals is much faster than that observed in humans, which makes it more difficult to identify variations and strong correlations in a relatively small population of animals, in which all cats are adults and there will be no major variations in the functional maturity of the OB. Similarly, a greater number of senior cats would allow a clearer conclusion to be drawn about the changes that occur in the OB in older animals.

**Figure 12:**
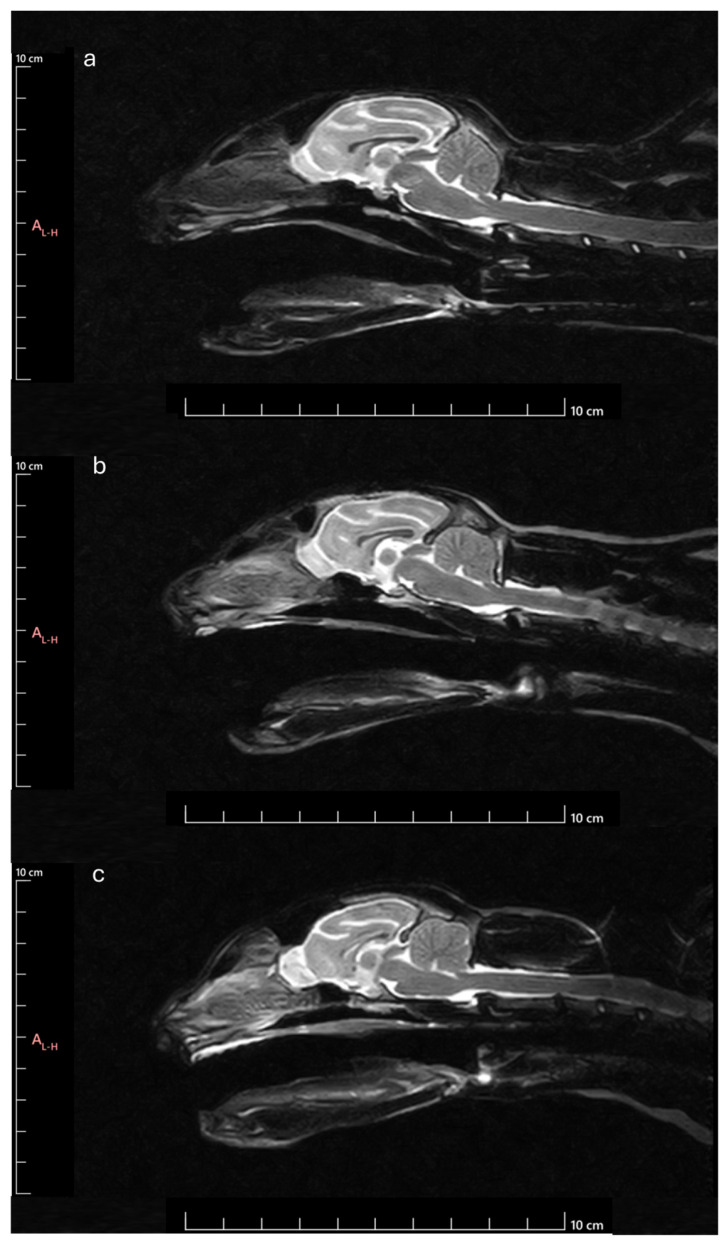
MRI Midsagittal T2-weighted images of animals with different head conformations: (**a**) animal classified as brachycephalic–ventral orientated and compressed OB with smaller OBSA, smaller OBA and smaller OBO; (**b**) Animal classified as mesocephalic-OB with intermediate orientation and OBSA, intermediate OBA and intermediate OBO; (**c**) Animal classified as dolichocephalic–dorsally orientated and globose OB with larger OBSA, higher OBA and higher OBO.

In summary, the results observed in the different variables indicate that brachycephalic cats tend to have OBs with a ventral orientation and establish smaller angles with the line that goes from the hard palate to the intercondylar notch of the foramen magnum, in comparison with dolichocephalic and mesocephalic breeds (Figure 12). These characteristics, shared with dogs, are related to the development of the skeleton of the head, which in these breeds results in premature closure of certain sutures of the head [10], leading to a shortened muzzle and rounded head and significant changes such as markedly reduced or absent frontal sinuses, shortening of premaxillary and maxillary bones, aberrant nasal conchae and lower height of the naso-osseal aperture [19,42]. Those generally promote the narrowing of the nasal and pharyngeal air passages while causing compression of the cribriform plate and reduction and flattening of the ethmoid fossae. Logically, the OB also suffers from this anatomical compression, appearing flattened, with a smaller cross-sectional area and oriented ventrally. The ventral orientation of the cribriform plate and OB is related to other conformational changes in the brain, such as the ventral orientation of the frontal lobes. Likewise, they are also associated with ventrally oriented ethmoid turbinates that may project into the airways and soft palate–epiglottis overlap, contributing to the complex BOAS patterns in these breeds [17,18]. This means that OB characteristics can be used to estimate the level of brachycephaly and the likelihood of developing associated complications in animals subjected to MRI.

This work has some weaknesses that should be considered, and which are explained by the preliminary nature of the study. The number of animals used is relatively small, and the uneven number of animals included in each cranial conformation group, although resulting from the selection of clinical cases following the inclusion criteria, makes it difficult to draw deeper conclusions and identify statistical differences and more unequivocal correlations. There is also no marked difference in the ages of the animals included in the different groups, which allows to identify small differences between young and adult animals but not draw deeper conclusions regarding senior animals and the possible atrophy of the OB secondary to phenomena of neurodegenerative atrophy. The weight of the animals was not considered, and to adjust for differences in the cranial dimensions measured, standardization relative to the cats’ weight could be beneficial. Determining the effective volume of the OB could provide an alternative method to better determine the dimensions of the OB in a complementary way to measuring the sagittal section area. Other elements that may influence the characteristics and cellular activity of the OB, such as the reproductive state of the animals, especially females, were also not explored. The review of the MRI images was performed by only one reviewer, which despite all measures taken to mitigate risk and the objective nature of the measurements performed, may raise questions about confirmation bias. To mitigate this factor, it will be important in the future to validate the methodology developed here using automatic measurement methods that do not require direct intervention from an operator.

Perhaps the most relevant limitation of the present work is the fact that it is based exclusively on a sagittal plane approach without considering the transverse plane and the information that can be obtained in a complementary way. Although the sagittal plane can provide valuable insights into OB orientation, angles, and cross-sectional area, it does not fully capture the structural complexities of the OB. The transverse plane is critical for achieving a more comprehensive understanding of the OB’s three-dimensional morphology and its integration within the cranial and intracranial architecture. For instance, the transverse plane would allow the assessment of bilateral symmetry or asymmetry in the OB shape and positioning, an aspect that is particularly relevant in the context of cranial conformational adaptations. Such asymmetries could be indicative of compensatory mechanisms linked to structural compression in brachycephalic animals or lateralized alterations associated with the development of certain pathologies. Moreover, the transverse plane analysis would offer the opportunity to evaluate the cross-sectional relationships of the OB with surrounding structures, including the cribriform plate, ethmoid bone, or the nasal cavity, and how these neighboring structures adapt together to the conformational and dimensional variations in the skull in the different cranial groups considered. These relationships could elucidate how bone compression or displacement affects not only OB morphology but also the olfactory pathways upstream of the rhinencephalon. In addition, the transverse plane could provide a foundation for volumetric analysis, which could significantly enhance the current approach. Measuring the OB’s effective volume, in combination with sagittal and transverse sectional data, would allow for a more accurate and multidimensional understanding of OB dimensions and their correlation with cranial conformations. This volumetric approach is particularly valuable in identifying subtle morphological changes, which might not be evident in a single plane but become apparent when analyzed in a comprehensive and integrative manner. The incorporation of the transverse plane could also strengthen the study’s relevance for clinical applications. Conditions such as BOAS and other cranial abnormalities often involve alterations that affect multiple dimensions of the OB and its surrounding structures. Transverse imaging would provide critical information to assess these changes and predict potential complications, not only for olfactory dysfunction but also for its contribution to respiratory compromise. It may also allow for more precise diagnostic imaging protocols, tailored to specific cranial conformations. Furthermore, integrating the transverse plane would enable the exploration of potential functional asymmetries within the OB, which could be relevant in the context of neurodegenerative diseases, metabolic conditions, or traumatic injuries. Functional imaging studies have demonstrated lateralized brain activity in response to olfactory stimuli in humans and companion animals [43,44], and transverse measurements could help establish whether this lateralization extends and is associated with structural asymmetries in the OB in cats.

This study represents an essential step in characterizing the sagittal morphology of feline OB, establishing relationships between its anatomical features and cranial conformations with unprecedented detail. Building on these findings, future studies should prioritize incorporating transverse plane exploration to enhance the anatomical understanding already achieved, particularly by revealing potential lateralized adaptations, volumetric relationships, and compensatory mechanisms associated with different head conformations. By expanding on this work, future research could provide a more comprehensive framework for understanding OB morphology and its clinical implications, particularly in the context of functional asymmetries observed in companion animals and particularly in cats.

## 5. Conclusions and Future Perspectives

This preliminary work represents the first attempt to morphologically and dimensionally characterize cats OB using a sagittal plane approach, determining the relationships with the cranial conformation and other head structures. It was established that animals with cranial conformations classified as brachycephalic have more compact OBs, with smaller cross-sectional areas, ventral orientation, and smaller angles established with the line that goes from the hard palate to the intercondylar notch of the foramen magnum. In contrast, animals classified as dolichocephalic have larger cross-sectional areas, dorsal orientation, and larger angles. Mesocephalic animals present an intermediate position. Males and younger adult animals have OBs with larger cross-sectional areas than females, and older animals have tendentially bigger cross-sectional areas than younger ones. The correlations identified, although lacking further validation and a new integrated approach with the findings obtained in the transverse plane, open doors for the use of this structure of the olfactory system and the central nervous system as an early indicator for the establishment of alterations of varied etiology, such as atrophy secondary to the development of neurodegenerative diseases in aging animals, alterations secondary to metabolic and infectious diseases and early indicators of risk for the development of complications related to BOAS in animals classified as brachycephalic. In the future, it will be important to carry out new studies with larger populations and apply a transverse plane approach to identify possible compensatory changes that may counteract the morphological variations described in this work. The methodologies can then be applied in animals with specific pathologies to validate their potential as a diagnostic tool, and even establish relationships with tests to assess the olfactory acuity of animals in vivo, to confirm the potential importance of OB and its imaging assessment in the clinical practice of companion animals.

## Figures and Tables

**Table 1:** Classification of animals included in the study considering their head conformation. SI, skull index; N, number of animals.

Head Conformation	SI	N	Sex	
**Brachycephalic**	>80	17	**Male**	8
			**Female**	9
**Mesocephalic**	75–79	9	**Male**	6
			**Female**	3
**Dolichocephalic**	<75	14	**Male**	9
			**Female**	5

**Table 2:** Results of measurements of the variables under study, considering the head conformation of the animals used, and respective statistical differences identified. OBA, Olfactory Bulb Angle; OBO, Olfactory Bulb Orientation; OBSA, Olfactory Bulb Sagittal Section Area. * corresponds to 0.01 ≤ *p* < 0.05, ** to 0.001 ≤ *p* < 0.01, *** to 0.0001 ≤ *p* < 0.001, and **** to *p* < 0.0001. nd—non defined.

Head Conformation	OBA (°)	Mesocephalic	Dolicocephalic	OBO (°)	Mesocephalic	Dolicocephalic	OBSA (mm^2^)	Mesocephalic	Dolicocephalic
**Brachycephalic**	64.99 ± 1.01	nd	****	12.79 + 0.55	nd	**	63.89 ± 2.55	nd	nd
**Mesocephalic**	68.28 ± 1.37		nd	13.95 ± 2.15		nd	66.35 ± 4.30		nd
**Dolichocephalic**	73.12 ± 1.01			21.18 ± 2.15			69.48 ± 1.89		

## Data Availability

The data that support the findings of this study are available from the corresponding author on request.

## Abbreviations

BOAS	Brachycephalic Obstructive Airway Syndrome
OB	Olfactory Bulb
OBA	Olfactory Bulb Angle
OBO	Olfactory Bulb Orientation
OBSA	Olfactory Bulb Sagittal Section Area
SI	Skull Index
SL	Skull Length
SW	Skull Width
